# Participatory Research to Build Narrative Power: Results From Survey Research to Support Community Organizing for Health Justice and Equity

**DOI:** 10.1111/1468-0009.70101

**Published:** 2026-06-03

**Authors:** YUSRA MURAD, KRISTINA MEDERO, CHLOE GANSEN, JASMINE SANDATE, MARISSA HALLO, SARAH E. GOLLUST

**Affiliations:** ^1^ University of Minnesota School of Public Health; ^2^ *Hubbard School of Journalism and Mass Communication* University of Minnesota; ^3^ Center for Health Progress Denver Colorado

**Keywords:** community organizing, health equity, narrative strategy, power, social determinants of health, survey research, collective action

## Abstract

**Policy Points**

- Community organizers addressing health issues use narrative strategy to shift public attitudes, build support for health‐affirming policies, and motivate collective action.
- Through a mixed methods research partnership with a power‐building organization, we evaluate how narrative power is leveraged to disrupt dominant ideas about the health care system—testing the impact of counter‐narratives grounded in the lived experiences of people most harmed by structural failures.
- Findings demonstrate that strategic messaging from community organizers about health policy issues has the potential to shift policy‐relevant beliefs, particularly those related to attributions of blame and intent to take collective action.

**Context:**

Building narrative power, a foundational strategy used in community organizing, involves dismantling dominant narratives that uphold inequity and constructing counternarratives that advance health equity and racial justice by reshaping how people make sense of the world. Yet, the mechanisms through which narrative power can influence public opinion and subsequent policy are seldom evaluated with research.

**Methods:**

In partnership with a power‐building organization, we developed and tested two counternarratives that challenge dominant framings of the US health care system. In this three‐phase study, we (1) conducted interviews with community organizers to explore their motivations and health care experiences, (2) iteratively developed two counternarratives reflecting the system's complexity and unaffordability, and (3) tested the counternarratives in a randomized experiment (*N* = 1,587), against a constructed dominant narrative and a no‐message control. Following exposure to an assigned narrative, participants completed a posttest questionnaire measuring the primary (policy perceptions and intentions to participate in civic actions to improve health care) and secondary (perceptions of causal attributions and blame) outcomes.

**Findings:**

Our results indicate the dominant narrative effectively individualizes blame for poor health and dampens motivation for civic engagement. Meanwhile, counternarratives rooted in the lived experiences and language of those most harmed by the system have potential to shift public beliefs, increase attribution for poor health to external factors vs. internal factors, and motivate some types of collective action.

**Conclusions:**

Our findings show the dominant narrative about health care does what health justice organizers argue it is designed to do: perpetuate harmful myths of individual blame for structural problems, shifting blame for health challenges away from powerful institutions. However, there is potential for counternarratives to reframe public perception and motivate collective action. Our study underscores the value of research partnerships between grassroots organizers and academic researchers in developing narrative power strategies.

For generations, grassroots organizing for health equity, racial justice, and health care system transformation has been a key avenue through which racially and economically marginalized communities have built power to achieve health and well‐being. In recent years, some academic and community‐based public health practitioners have turned more attention to the role of *narrative power* in grassroots and community organizing. Narrative power can be defined as “the ability to make the foundational stories we tell—the stories about how things work, our sense of history, who and what matters, and our relationship to one another and the planet—the main stories people use to make sense of the world over time.”^1^, ^2^

A key element of building narrative power is challenging dominant narratives that have permeated the imaginations of the public and defined the boundaries for what is considered *feasible* or *possible* with respect to policymaking that advances health and racial justice.^3^ In the context of health care, dominant narratives tend to focus on the exceptionalism and innovativeness of the American system, while minimizing the immense financial burdens, inequities, and complexity associated with these supposed advancements. Another related dominant narrative links personal responsibility and deservingness of health care, making health care access conditional on individual effort, characteristics, or behaviors. This narrative in practice and policy allows the connection between health insurance and employment to seem rational or commonsense (eg, employer‐sponsored health insurance or policies to require employment as a condition of Medicaid) and policy efforts to disrupt this relationship implausible or politically fraught.^2^ Collectively, these dominant narratives implicitly—and sometimes explicitly—blame poor health outcomes on decisions made by individuals, particularly those who belong to marginalized communities.^4^ This “poor choices” framework diverts attention from structural problems and the people and institutions that derive financial benefit, status, and power from the existing system, thus upholding the entrenched, discriminatory practices that reinforce exclusion.^5^ Theory from multiple disciplines suggests that by misattributing the cause of poor health outcomes to individual‐level characteristics and behaviors (ie, internal causal attributions), dominant narratives about the health care system may reduce public support for policies with potential to address structural drivers of inequity.^6^, ^7^, ^8^

Community organizing is one pathway through which people can cocreate new stories—or counternarratives—that draw on lived experiences and power analysis to shift and dismantle dominant narratives that uphold oppression.^5^ Organizers demonstrate the potential of such counternarratives to shift social norms and public worldviews, where worldviews are understood as a component of the upstream structural determinants of health influencing policies, practices, and investments that shape conditions on the ground.^9^ Indeed, the collective development of counternarratives that “empower and give agency” to those in the margins^10^ is a key function of power‐building organizations, rooted in the long‐standing traditions and legacy of Black, Brown, and Indigenous people‐led social movements,^11^ which have shifted public consciousness away from a sole focus on individual behaviors and outcomes and toward disrupting the structures and systems that make people sick. As a specific example, People's Action, a national network of power‐building organizations across 29 states, leads a “Health Care for All” campaign. Within that campaign, organizers use specific language to challenge the dominant narrative that only some people deserve health care as a function of their individual effort, behaviors, or work. Instead, the campaign's counternarrative consistently emphasizes that “Health care is a human right” and “We all deserve lifesaving care and medicines, when we need them” in order to win universal health care, universal access to lifesaving medicines, and lower drug prices and hold corporations accountable for putting profit over people.^12^, ^13^

While narrative power is growing in recognition as a key organizing strategy, the *mechanisms* through which narrative power influences public perceptions (and ultimately policy change) are seldom tested through evaluative paradigms conventionally used by researchers.^14^, ^15^ To examine how organizing narratives shape public opinion, we established a two‐year partnership with a multiracial, member‐led, power‐building organization based in Colorado: Center for Health Progress (CHP). CHP's grassroots organizing is led by community members directly impacted by the health care system, including health care workers, undocumented and uninsured people, and working‐class families. CHP defines itself as a base‐building organization, meaning the organization's priorities flow from this “base” of community members who are connected with one another and united in a common identity and shared understanding of the root causes of adverse health care outcomes. While we recognize base building as core to fostering shared identity and a robust relational foundation, we focus here specifically on the community organizing phase of the process. Through organizing, individuals or groups within the “base” are transformed into actors who can strategically and effectively pursue shared goals in the public domain by taking collective action.^16^ We designed and conducted the present study alongside CHP's communications organizing team, which includes organizing staff and community leaders recruited to focus on narrative power.

This collaboration was rooted in the overarching question motivating CHP's communications organizing team: *How do we counter the dominant narrative about health in a way that resonates with people, empowers them to see themselves in a counternarrative, and mobilizes collective action?* To answer this question, we worked collaboratively to test the impact of counternarratives—anchored in the community leaders’ lived experiences, values, and visions—on people's attitudes and beliefs about the health care system. This methodological choice represents divergence from traditional peer‐reviewed literature conducting message‐testing experimental research (see, eg, Niederdeppe and colleagues^17^ for a review) in which researchers might craft narratives or messages based on their *perception of* the experiences of directly impacted communities, but often without authentic or sustained partnerships. Our study team is one of a small number of documented partnerships between public health researchers and community organizers.^18^

## Background on US Health Care System Problems

Although the United States invests more resources into health care than any other country in the world, it consistently underperforms on key measures of health care system efficacy, particularly access to care, equity, and health outcomes.^19^ Compared to other high‐income countries, Americans face the most avoidable deaths, the lowest life expectancy overall,^19^ and staggering disparities across racial groups.^20^ The ballooning cost of care paired with administrative complexity force patients to navigate enormous barriers to accessing and affording health care, while health care companies’ profits soar.^21^ Significant cost‐sharing requirements in private insurance leave millions of working people “underinsured” and unable to access care due to out‐of‐pocket costs, despite paying hefty monthly premiums, leading them to delay or forgo necessary medical care.^19^ Despite gains in insurance coverage attributable to the 2010 Affordable Care Act, 26 million Americans are still uninsured.^22^

Rising health care costs and negative experiences for patients are driven in part by the worsening complexity of the health care system. For example, insurers may require providers to demonstrate that medical treatments recommended for patients are necessary—a process fraught with opportunities for error, delays, administrative complications, and bias.^23^ Survey research suggests the majority of insured people have problems utilizing their coverage, especially those in fair or poor health, those who need mental health services, and/or those who use the health care system the most.^24^ Unexpected bills, language barriers, and lack of in‐network availability are just a few of the complications faced by insured adults, who overwhelmingly support policies to bring down the cost of care and make health insurance easier to navigate.^24^ But, the likelihood of meaningful policy action for patient protection at the federal level is low as a result of the current Republican leadership's opposition to regulation.^25^ Community organizing around these policy issues is one important strategy for making change, often at the local level. CHP, like other power‐building organizations, is focused on empowering people most harmed by the health care system to help set the agenda for change and hold systems accountable.

## Background on Power‐Building Organizations and Narrative Strategy

While direct collaborations with power‐building organizations are still rare in mainstream public health research,^18^ pockets of critical public health scholarship have focused more attention in recent years to the role of *power* in shaping, maintaining, and reinforcing health inequities, and relatedly, the capacity of *power‐building organizations* to shift power structures, impact public attitudes about a range of health policy issues, and drive policy agendas through organizing and advocacy toward health justice.^3^ Health justice, Michener writes, is a process that “entails transforming existing economic and political institutions to make them more inclusive, responsive, and accountable” ^26^ ^(p657)^; health justice is closely aligned with health equity but has a sharper focus on *building power* among those most exposed to health inequity and *breaking the power* of those who stand to benefit from it. At the core of building power for health justice, Michener articulates, is “cultivating the political capacity of people who are disproportionately harmed by health inequity, and who therefore have the most at stake.”^26^ ^(p656)^ Building partnerships with grassroots organizers, and pursuing projects that strengthen organizing campaigns seeking to improve the material conditions of populations disproportionately harmed by structural inequities, is one strategy through which the public health discipline can contribute to ongoing efforts to shift power from economic and political institutions to the communities that are not only most impacted by the existing system but also leading efforts to demand more equitable futures.^26^, ^27^ Such partnerships can also challenge deficit‐based frameworks, which portray communities of color as having inherent problems needing to be fixed and may reinforce myths of personal responsibility, by foregrounding community knowledge, agency, and leadership.^28^

Community organizing for social change is rooted in a fundamental belief in “community power,” defined by Pastor and colleagues as follows:

The ability of communities [most impacted by structural inequities] to develop, sustain, and grow an organized base of people who act together through democratic structures to shift public discourse, set proactive agendas, influence who makes decisions, and cultivate ongoing relationships of mutual accountability with decision makers that change systems [and advance health equity].^27^ ^(p3)^

Note that the brackets in the quote were added to the original to denote that Pastor's definition explicitly favors equity goals and those most impacted. This definition is anchored in Lukes's^29^ “three dimensions of power” framework, a contemporary conceptualization that has informed local and national organizing efforts (eg, Grassroots Power Project^30^) through identifying *observable*, *hidden*, and *invisible* power as three interrelated “faces” of power. Where “observable” and “hidden” power encompass, for example, policy campaigns and the creation of long‐term alliances, “invisible power” concerns public discourse, narratives, and understandings or beliefs about society.^27^ Through dominant narratives amplifying myths of individual causal attribution of poor health or the exceptionalism of US health care, health care organizations consolidate, normalize, and wield *invisible power* to drive policy actions and decisions that advance or maintain inequitable power relations.^3^, ^31^, ^32^ In contrast, counternarratives rooted in critical analysis of institutional power and economic interests have the potential to challenge how audiences make sense of the problems they experience with the health care system and the solutions they associate with addressing these problems.

It is worth noting that community organizing is not inherently in service of equitable outcomes; throughout history, groups have organized and built narrative power in ways that exclude marginalized populations, protect concentrated wealth, and uphold systemic hierarchies. However, in the present study, we situate community organizers in building cross‐race, cross‐class coalitions, with an explicit orientation to health equity, in building narrative power to advance public health. In this context, community organizing is a critical site of political struggle, where organizers contest dominant narratives, negotiate and develop counternarratives, and build narrative power to shape public worldviews and motivate collective action.^33^ By centering personal relationships and investing time and resources into learning people's stories, organizers generate knowledge about how the structural determinants of health harm communities. This process recognizes that narrative power is most effective when embedded in the relational and emotional dimensions of empowerment that drive collective action.^34^, ^35^ In other words, when narrative interventions are approached not only as communication strategies but are also contextualized and integrated within a broader framework attending to the multiple dimensions of power (eg, deployed through organized bases that exert political and economic pressure), they can be key elements of subverting harmful power relations and driving social change.^33^ Within this multifaceted developmental process, organizers develop campaign strategy and mobilize diverse constituencies around shared visions for more equitable redistribution of health‐sustaining resources in which the development of and sharing of counternarratives are key.

This work is anchored in the assumption that the effects of counternarratives, particularly those born in cross‐class, cross‐racial coalitions, on perceptions and behavior “make it a promising lever for health equity‐focused intervention.”^33^ ^(p2)^ Many organizers rely on a framework for building counternarratives called “Race‐Class Narrative” (RCN).^36^ RCN is a messaging strategy with growing evidentiary support; it intentionally intertwines racial justice and economic justice to undermine traditional “divide‐and‐conquer politics,” aiming to motivate collective action to dismantle structural inequities and advance the shared interests of a multiracial working class.^14^ A few dimensions are critical to this messaging strategy: opening with explicit mention of shared values, referencing race and class explicitly, discussing problems and solutions only after naming shared values, and identifying the “villain” or antagonist that causes harm.^37^

## Background on Survey‐Based Experiments and Goals of the Present Study

In survey‐based experiments, randomly assigning respondents to read distinct messages (eg, control and treatment conditions) is an established method for studying message effects.^38^ This approach has been widely applied in health policy communication research, offering valuable insights for journalists, practitioners, and communicators regarding how specific messages can shift policy‐relevant beliefs on topics as diverse as opioid use, adverse childhood experiences, mental health, and Medicaid.^39^, ^40^, ^41^, ^42^ For example, Gollust and colleagues^39^ used this method to illustrate how messages framing the consequences of adverse childhood experience in terms of economic impact can drive stronger public support for policy strategies, compared to messaging focused on other types of consequences. Taken together, research on the effects of health policy messaging from these studies suggests even relatively subtle differences in message framing have potential to shift public beliefs, particularly those related to policy support or attributions of blame.

Though this method is common in health communication research, treatment exposures are typically created by academic researchers rather than codeveloped with those directly affected or positioned to apply the findings in practice. Further, it is often infeasible for community‐based organizations to conduct this research, considering the costs of data collection and analysis. Research rigorously testing the effects of messages informed by the perspectives of community organizers, particularly those who have been harmed by the health care system, may provide deeper insights and recommendations that could, in turn, more effectively resonate with the public to influence beliefs and policy support.

There are a few other notable limitations of conventional approaches to studying message effects in academic research. For one, the outcomes explored in many framing studies are generally related to beliefs and policy sentiment, as opposed to more politically meaningful measures of intent to take political action.^17^ In addition, much of the existing research examining messaging strategies related to racial equity, in particular, relies on short messages (eg, a phrase or a few sentences), which can dilute or compress key messages, vs. longer, detailed narratives telling a story to help readers more fully understand the stakes of an issue.^17^ Finally, samples used in such studies often consist of participants who belong to advantaged demographic groups (ie, White, and/or higher income and education levels) instead of people who have experienced historical or contemporary disadvantages (eg, colonialism, racism, economic inequality).^17^, ^43^

The present study addresses these gaps by testing the effects of counternarratives on key outcomes identified in partnership with a power‐building organization. Specifically, a preregistered survey‐based experiment assessed how dominant narratives and counternarratives about the US health care system impact people's intent to participate in actions to improve the system, and their perceptions of causal attributions and blame.

## Methods

This study consisted of three stages, all conducted in collaboration with the CHP communications organizing team: (1) conducting qualitative interviews with CHP community leaders, (2) codesigning counternarratives to test using leaders’ language and lived experiences and identifying key outcomes to assess, and (3) fielding a quantitative survey to examine how different narratives influenced outcomes of interest. Throughout the project, we met regularly and shared interim and final results with the CHP staff and community leaders. This mixed‐methods research protocol allowed for iterative feedback from CHP staff and community leaders at every stage of research development through dissemination.

### Phase 1: Qualitative Interviews to Develop Narrative Themes

In the first phase, we conducted 1‐hour, semi‐structured interviews with seven members of CHP's communications team—a cohort of leaders developing CHP's narrative strategy with staff. The interview guide was cocreated with staff and leaders. All participants identified as women or nonbinary. Five were parents, four identified as non‐White, and two worked in health care.

Interviews explored (1) motivations for power‐building and health care advocacy, (2) challenges with the US health care system and perceptions of responsibility for these challenges, and (3) visions of an equitable, person‐centered health care system. The interviews were transcribed via Otter.ai in real time, with edits and revisions made following the interviews to identify transcription errors. Field notes were taken immediately after each interview. The research assistant responsible for interviews documented key themes for the purpose of sharing and co‐interpreting with the research team and CHP.

Once interviews were complete, the research team developed a preliminary coding scheme based on field notes and discussions with community leaders. We established qualitative procedures, following the guidance of Miles and colleagues,^44^ to conduct the qualitative analysis, focusing on the identification of emerging themes and comparison of themes across participants’ roles and relationship to the health care system. The team‐based, inductive approach was used to finalize the codebook and encompass all emergent themes. All transcripts were double‐coded in Dedoose by two team members. Discrepancies in coding were discussed until agreement was reached. Appendix A describes the major themes of the interviews. In brief, the most salient themes interviewees shared were that they experienced harm from navigating health care, stemming from the high costs of care and the burden of coordinating a complex and disorienting system while navigating illness. They felt disempowered in their interactions with the system, and the experiences overall left them cautious and avoidant of care. They articulated systematic oppression within the system, perceiving that they are treated differently based on their racialized identity or English literacy. Finally, they identified the profit motive within health care as a major contributor to these challenges, and they wished for a new system where they felt greater agency. We shared these themes with the organizing team to ensure the validity of the results, using a participatory process where the team could identify how strongly they resonated with each theme (Appendix A).

### Phase 2. Codesign of Narratives and Outcomes Identification

#### Counternarratives

In the next codesign phase, we used the themes from phase 1 to identify key narrative elements for our survey experiment, anchored in the RCN framework (ie, begins with shared values that are explicitly across racial groups and social classes; naming the problem and its “villain” or architects).^37^ Relying on the team's expertise in health communication, the RCN framework, and the leaders’ training in community organizing, we collectively identified a set of narrative elements we considered important to construct a persuasive narrative, including a statement of shared values, a problem statement (with consequences), a statement naming the villain or antagonist causing the problem, an acknowledgment of the myths or counterarguments endorsed by the antagonist (ie, a form of inoculation),^45^ a statement of a vision for change, and a call to action.

In drafting the counternarratives consisting of each of these narrative elements, we had three main priorities. The first was ensuring the language used in the messages was consistent with how CHP staff and community leaders authentically spoke about challenges with the health care system during organizing conversations. To do so, whenever possible, we used language directly from leader interviews to ground the narratives in their voices. The second priority was choosing narrative elements seen by *CHP community leaders* as having clear potential to shift public attitudes, beliefs, and intentions to take action (vs. exclusively relying on existing literature). The third was to avoid jargon and write as directly as possible. For example, we described the concept of being a “self‐advocate” as the unfair feeling of having “to be the one who knows how all the pieces [of the health care system] fit together” rather than using that language. Our methodological choice to build the counternarratives tested in our study from stories shared by CHP leaders reflects our partnership's approach to narrative interventions that are grounded in the leadership, lived experiences, and relational power of those most impacted, rather than treated as isolated messaging tools.

The final counternarratives focused on the two primary issues elevated in interviews with CHP leaders (and endorsed when we shared results back) as most important: unaffordability and complexity of care. These issues are widely shared, as public opinion evidence consistently reveals that unaffordability and complexity of the health care system are two of the most important issues facing Americans.^46^, ^47^

#### Dominant Narrative

The dominant narrative about health care in the United States was crafted by reviewing prominent health care organization websites, communications, and messages identified from CHP's campaign research. We discussed several drafts of the dominant narrative with CHP leaders, with a particular eye to capturing messages about the exceptionalism of the US health care system and myths of individual blame that members of CHP identified as most harmful to them as individual patients as well as in their organizing efforts. Feedback from CHP leaders was incorporated into the final version. The full narratives are displayed in Appendix B.

Once the narratives were finalized, we used a deliberative process with the leaders to identify and rank the primary outcomes of interest relevant to CHP's organizing goals—if and how narratives shape people's intent to participate in actions to improve the system. We also identified secondary outcomes—if and how narratives shape perceptions of causal attributions (ie, who is to blame for the problems with health care in the United States). As described below, we then identified established survey measures to capture these key outcomes of interest.

### Phase 3. Fielding the Survey

We next fielded a within‐subject survey experiment, with an instrument that included the narratives developed and outcomes identified from phase 2. Participants were recruited from the Prolific platform, a survey panel service. US adults who spoke English were eligible to participate, and we aimed to recruit an equal number of people who identify as male or female. We conducted an a priori power analysis with the expectation of a small to moderate effect size (η^2^ = 0.20) for a fixed effect (with an alpha level of 0.05 and power of 0.80). Therefore, we aimed to recruit 1,600 participants to ensure a sample size of about 400 individuals per group.

After agreeing to participate in the survey, participants were randomized to view either the no‐exposure control condition or one of three treatment conditions that differed only in the narrative shown: the dominant narrative, an unaffordability counternarrative, or a complexity counternarrative. Participants in the dominant narrative condition were told the message was adapted from materials shared by a large health care system in the midwestern United States. Those in the counternarrative conditions were told the message was written by a community member who volunteers for a nonprofit advocacy organization, has a lot of experience navigating the health care system, and has struggled to get care for herself and her family. Following exposure to their assigned narrative, participants completed a posttest questionnaire measuring the primary and secondary outcomes (listed below). Those in the control condition did not see a narrative and simply proceeded to the questionnaire. The full description of our hypothesis, measures, and analysis can be found preregistered on OSF (https://osf.io/836ax/). Data were collected from March 22 to March 24, 2024 (after completing a small pretest in January 2024).

#### Primary Outcome Measures

*Primary outcomes* were intentions to participate in actions to improve health care, with three measures: (1) policy agenda setting, (2) collective action intentions, and (3) social justice behavioral intentions.

*Policy agenda setting* was measured by averaging responses to two items adapted from the literature^39^: “How important do you think it is for the government to do something to make health care more affordable for all people living in the United States?” and “How important do you think it is for the government to make health care less complicated for all people living in the United States?” on a five‐point Likert scale from “Not at all important” to “Very important” (*α = *0.87, *M = *4.45, *SD *= 0.91).

*Collective action intentions* were measured by participants indicating their likelihood of engaging in ten activities, in the next few months, that could “influence the government or health care system to take action to improve health care,” on a five‐point Likert scale from “Extremely unlikely” to “Extremely likely.” The list of activities was based on prior measures of collective action intentions^15^, ^39^ and included activities such as “Vote for or against a candidate for public office because of their position on health care policies” and “Participate in a rally or protest to improve the health care system.” Responses to the ten items were averaged to create a composite intention score (*a *= 0.93, *M *= 2.56, *SD *= 0.96). We also examined items individually, given their conceptual variability.

*Social justice behavioral intentions* were measured as the average of four items adapted from the literature^48^ that asked the extent to which participants would “engage in activities that will promote a more fair health care system” on a five‐point Likert scale from “Strongly disagree” to “Strongly agree.” Activities included “I will do my best to ensure that all individuals and groups have a chance to speak and be heard” and “I intend to engage in activities that will promote a more fair health care system” (*α *= 0.91, *M *= 3.50, *SD *= 1.03).

#### Secondary Outcome Measures

*Secondary outcomes* were perceptions of causal attributions and included two measures: (1) blame attributions and (2) a single item measure of internal vs. external causal attribution.

*Blame attribution* for the unaffordability and complexity of the system was assessed for seven entities: patients, health care executives, the federal government, the food and beverage industry, the pharmaceutical industry, the medical device industry, and the health care industry (1 = Not at all, 5 = A great deal).^49^ Scores for health care executives, the pharmaceutical industry, the medical device industry, and the health insurance industry were averaged to create a “medical industry” causal attribution score (*ɑ *= 0.78, *M *= 4.15, *SD *= 0.75). Scores for the remaining entities were treated as single items.

With respect to *internal vs. external causal attribution*, participants were asked to select which of the following two statements more closely aligned with their own views: “The biggest reason people in America become unhealthy is because they make poor choices that affect their health” or “The biggest reason people in America become unhealthy is because things outside of their control affect their health.”^50^ Selecting the first statement was indicative of holding an internal causal attribution (44.9%), and the second was indicative of an external causal attribution (54.9%).

#### Measures of Potential Moderators

Also included in the survey were measures for several potential moderators, including *income*, *cost barriers to health care*, and *partisanship*, all subgroups of particular interest to the CHP organizers. We were interested in whether the narratives would be particularly effective among working‐class people and those with adverse experiences with the health care system, and how they would resonate across partisan identity groups.

Income was measured as a self‐report of total household income per year (in categories of $10,000). Partisanship was measured via a single item assessing respondents’ affiliation with the main political parties in the United States: “Generally speaking, do you think of yourself as a Democrat, a Republican, an Independent, or what?”^51^

To measure barriers to health care, we adapted an item from a Perry Undem^46^ research firm survey that asked participants whether they had experienced nine adverse scenarios in the last year (eg, “Self‐treated with home remedies or over‐the‐counter medications,” “Delayed or skipped getting mental health care,” “Left a prescription at the pharmacy because it was too expensive”) due to health care costs. We constructed an ordinal variable with three categories: low experience with health care barriers (zero to two barriers), moderate experience (three to five barriers), and high experience (more than five barriers).

### Analysis

Our preregistered, central hypothesis is that the counternarratives will increase intentions to participate in actions to improve the health care system (our primary outcome measures). Specifically, we expected participants in the unaffordability and complexity counternarrative conditions to report stronger intentions related to policy agenda setting (H1a), collective action (H1b), and social justice behaviors (H1c), compared to those in the control or dominant narrative condition.

Our secondary hypothesis is that the counternarratives will shift perceptions of causal attributions for problems with the health care system. Specifically, we expected participants in the unaffordability and complexity counternarrative conditions to attribute more blame for problems with health care to systemic factors over individual factors, compared to the control or dominant narrative condition (H2).

The first step in the analysis was exploring data for missingness for our primary and secondary outcome variables. We found little missing data and made no adjustments to the sample. In addition, we examined two measures of time: time to complete the survey and time spent on the message to which the respondent was assigned. There was no indication of “speeders” (ie, spending less than 2 minutes for the whole survey, spending less than 5 seconds on the messages); thus, no observations were dropped from the analysis. Finally, no demographic (eg, race, gender) group was observed to be significantly maldistributed in any one condition (*P* > .05).

To test our hypotheses, we assessed mean differences across the four main experimental conditions (control/no narrative, dominant narrative, unaffordability counternarrative, and complexity counternarrative) on each primary and secondary outcome measure using analyses of variance (ANOVAs). To compare the four conditions to one another when the ANOVAs demonstrated significant differences across groups, we used Tukey's HSD (Honestly Significant Difference) post hoc pairwise comparisons (given the multiple comparisons tested). *P*‐values less than .05 were considered statistically significant, although in a few instances, we report findings for group differences between *P *= .05 and *P *= .09 to illustrate trends.

Additional exploratory analyses examined evidence of moderation of the effects of the four message conditions by income, partisanship, and barriers to health care. For these analyses, we estimated linear regression models with interaction terms with these select individual difference variables to examine whether the effects of the narratives on the primary outcomes differed for groups defined by these characteristics. Income was dichotomized to represent participants above and below the national median of income (ie, about $80,000).^52^ Barriers to health care was an ordinal variable that was treated as continuous in the interaction analysis. Partisan identity was a categorical variable with three categories (Democrat, Republican, Independent, excluding those who identified “another party”). (Sensitivity analyses with partisan groups defined with “leaners” included in the party groups vs. as Independents are presented in Appendix C, Table C.3). We then illustrated the interaction findings using predicted means. All analyses were conducted using Stata 17.

## Results

The total number of participants in our study was 1,587 (see Table 1). Although the sample was not nationally representative, the sample demonstrated moderate diversity (Table 1). For example, 24% of participants were 18‐29 years old; the median age was 38. About half (49.3%) of participants identified as male; less than half identified as Democrat (45.9%) and one in five as Republican (20.2%). Participants were predominantly White (62.3%) and had completed at least 4 years of college (54.7%), while 13.5% had a high school degree or less. Eighty percent reported at least one experience with cost‐related barriers to health care. On average, participants—particularly low‐income respondents—reported having about three experiences with barriers to health care, predominantly (1) using home remedies, (2) delaying dental care, and (3) delaying medical care.

**Table 1:** Demographics of Study Participants (*N* = 1,587) vs. US Population

Variable	*N* (%)	US Demographics^c^
Age	*M* = 40.8 (18‐94)	Median = 39.1
Gender		
Woman	762 (48%)	50.5%
Man	783 (49.3%)	49.5%
Non‐binary	29 (1.8%)	NA
Race		
White	989 (62.3%)	57.5%
Black	178 (11.2%)	13.7%
Asian	183 (11.5%)	6.7%
Latino/Hispanic	130 (8.2%)	20%
Mixed	107 (4%)	3.1%
Education^d^		
Less than high school	8 (0.5%)	10.1%
High school or GED	206 (13%)	27.3%
Some college, no degree	334 (21%)	18.5%
Associate's degree	171 (10.8%)	7.9%
Bachelor's degree	599 (37.7%)	21.6%
Master's degree	214 (13.5%)	NA
Professional or doctoral degree	55 (3.5%)	NA
Income		Median = $78,538^e^
Less than $30,000	418 (18.8%)	
$30,000 to $39,999	151 (9.5%)	
$40,000 to $49,999	130 (8.2%)	
$50,000 to $59,999	146 (9.2%)	
$60,000 to $69,999	122 (7.7%)	
$70,000 to $79,999	135 (8.5%)	
$80,000 to $89,999	89 (5.6%)	
$90,000 to $99,999	115 (7.2%)	
$100,000 to $149,999	224 (14.1%)	
Greater than $150,000	174 (11%)	
Political affiliation^a^		
Democrat	736 (46.4%)	46%
Independent	502 (31.6%)	NA
Republican	327 (20.6%)	47%
Experience with health care barriers^b^		
Low	668 (42.1%)	NA
Moderate	549 (34.6%)	NA
High	352 (22.2%)	NA

Abbreviation: NA, not available.

^a^ Democrats and Republicans include participants who reported they “lean” Democrat or Republican; Comparative data from Pew Research Center^53^ based on 2024 data.

^b^ Barriers to health care were measured categorically: 0 = low experience with health care barriers (0‐2), 1 = moderate experience with health care barriers (3‐5), 2 = high experience with health care barriers (5 or more).

^c^ US demographics come from the US Census Bureau from July 2024.^54^

^d^ Data based on US citizens 25 years of age or older. Data are not reported using the same increments, but 13.8% have a master's degree or higher.

^e^ Income increments not reported across data sources.

### Effects of Narratives on Intentions to Participate in Actions to Improve the Health Care System

#### Policy Agenda Setting

Among the four main experimental groups, respondents in the control group reported the highest perceived importance of government action, while those who read the dominant narrative reported the lowest. However, the differences across the four groups were not statistically significant (F[3, 1,584] = 0.67, *P* > .05); thus, our H1a prediction was not supported. Given that overall perceptions of importance were high across groups (*M *= 4.45), the elevated baseline support for government action may have limited the extent to which the experimental manipulation could shift responses.

#### Collective Action Intentions

**Figure 1:**
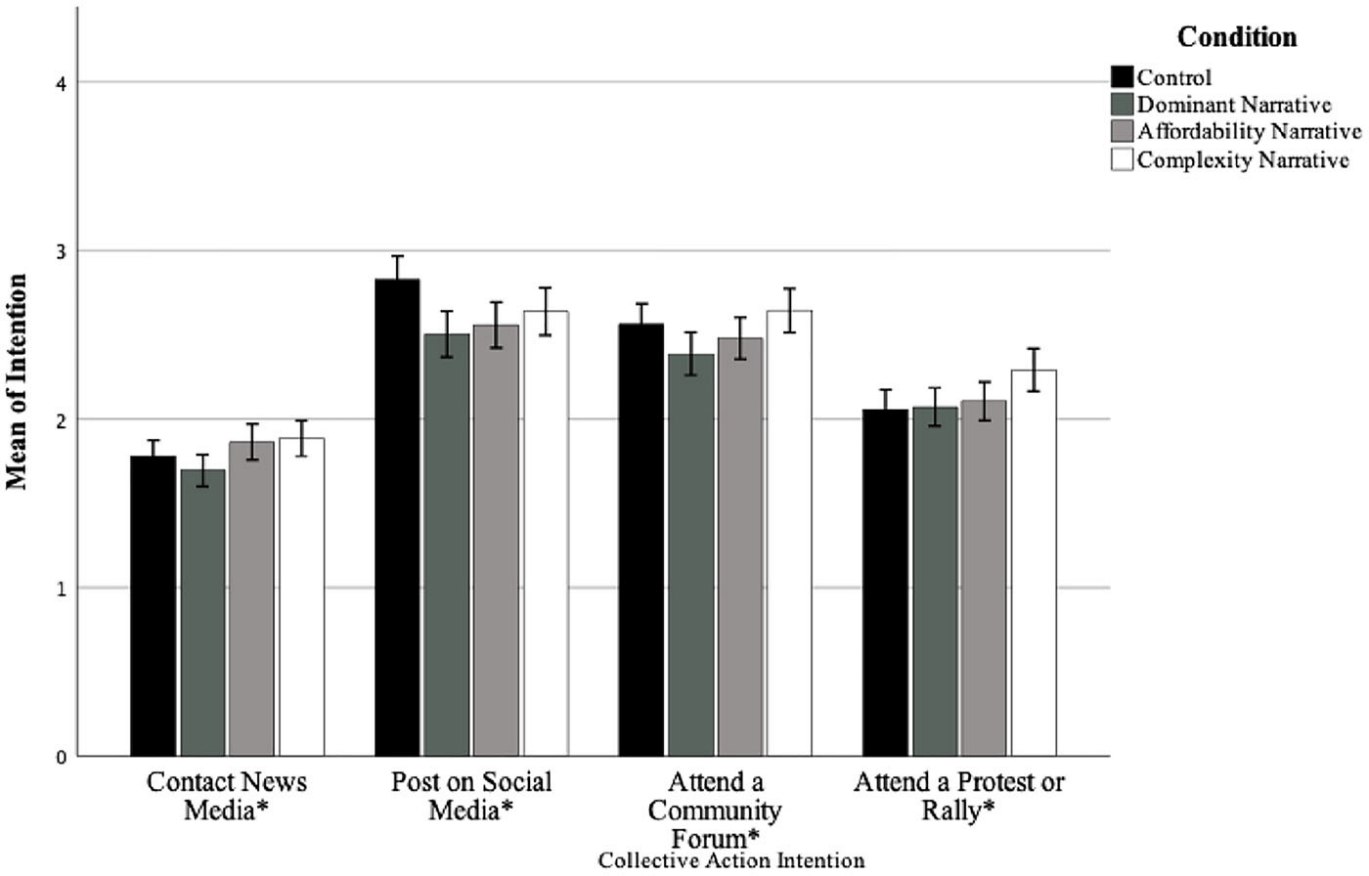
Means of Collective Action Intentions Across Experimental Conditions [Colour figure can be viewed at wileyonlinelibrary.com] * Significant differences across all conditions for these four outcomes; pairwise differences are reported in the text.

When all ten responses were averaged, there were no significant differences in outcomes across the four message conditions (F[3, 1,572] = 1.97, *P *= .12). However, we also examined items individually, given their conceptual variability. Although most of the relationships were insignificant, four variables were found to have significant or marginally significant differences across the four conditions (shown in Figure 1).

First, there was a marginal difference across the four groups in participants’ intent to contact a newspaper, television station, or radio talk show about their concerns about the health care system (F[3, 1,583] = 2.49, *P *= .059). Specifically, post hoc pairwise comparisons showed that participants exposed to the complexity counternarrative were more likely to contact the media than those exposed to the dominant narrative (Mean [M] Difference = 0.18, SE = 0.07, *P *= .062).

Second, there was a significant difference across the four groups in participants’ likelihood of posting a message on social media about their beliefs about the health care system (F[3, 1,583] = 4.37, *P* = .0045), although the post hoc pairwise comparisons showed the significant differences were not between the hypothesized groups: participants in the control condition were more likely to post a message on social media than those exposed to the dominant narrative (M Difference = 0.33, SE = 0.10, *P *= .004) or unaffordability counternarrative (M Difference = 0.27, SE = 0.10, *P *= .027).

Third, there was a small but statistically significant difference across the four conditions in participants’ intent to participate in a community forum (F[3, 1,583] = 2.88, *P* = .0347). Post hoc pairwise comparisons showed that the complexity counternarrative was the only condition that was significantly different from the dominant narrative (M Difference = 0.25, SE = 0.09, *P *= .027); participants exposed to the complexity counternarrative were more likely to participate in a community forum than those exposed to the dominant narrative.

Last, there was a small but statistically significant difference across the four groups in participants’ intent to participate in a rally or protest to improve the health care system (F[3, 1,580] = 3.03, *P *= .0284). Post hoc pairwise comparisons showed that participants exposed to the complexity counternarrative were more likely to report they would participate in a rally compared to the dominant (M Difference = 0.21, SE = 0.09, *P *= .064) and no‐exposure groups (M Difference = 0.23, SE = 0.08, *P *= .038). Thus, H1b was partially supported with regard to the intention of contacting the media, attending a community forum, and attending a rally, among those who read the complexity counternarrative.

#### Social Justice Intentions

There were significant differences across the four groups regarding intent to participate in social justice activities (F[3, 1,578] = 4.14, *P* = .0062). Respondents who were exposed to the dominant narrative reported significantly lower social justice intentions compared to respondents exposed to the control group (M Difference = 0.25, SE = 0.07, *P* = .004), and those in the complexity counternarrative group reported marginally lower social justice intentions compared to the control group (M Difference = 0.18, SE = 0.07, *P* = .063), providing some support for H1c.

### Effects of Narratives on Perceptions of Blame and Internal/External Causal Attributions

#### Blame Attributions

Participants were asked about the extent to which the following entities should be blamed for the costs and complexity problems in the health care system: patients, the medical industry (health care executives, the pharmaceutical industry, the medical device industry, and the health insurance industry), and the federal government. Significant results are reported in Table 2.

**Table 2:** Blame Attributed to Patients, Medical Industry, and Government Across Experimental Conditions

Condition	Mean (SD)	Diff. from control (*P*‐value)	Diff. from dominant (*P*‐value)
Patient blame			
Control	2.33 (1.11)	N/A	.246
Dominant narrative	2.48 (1.20)	.246	N/A
Unaffordability counternarrative	2.05 (1.09)	.004	<.001
Complexity counternarrative	2.13 (1.15)	.073	<.001
Medical industry blame			
Control	4.15 (0.73)	N/A	.229
Dominant narrative	4.05 (0.70)	.229	N/A
Affordability counternarrative	4.2 (0.77)	.811	.029
Complexity counternarrative	4.21 (0.79)	.692	.016
Federal government blame			
Control	4.01 (0.96)	N/A	.233
Dominant narrative	3.88 (1.06)	.233	N/A
Affordability counternarrative	4.12 (0.94)	.448	.005
Complexity counternarrative	4.10 (0.95)	.595	.010

We used Tukey's HSD post hoc pairwise comparisons to test differences in means, given multiple comparisons.

There were significant differences across the four groups in blame assigned to patients (F[3, 1,581] = 11.16, *P *< .001). In line with our H2 prediction, post hoc pairwise comparisons showed that both counternarrative conditions were significantly different from the dominant narrative, which increased blame attributed to patients, compared to the unaffordability and complexity counternarratives (*P *< .001 in both cases). Additionally, exposure to the unaffordability narrative significantly decreased blame attributed to patients compared to the no‐exposure group (*P *= .004).

There were also significant differences across the four groups in blame assigned to the medical industry (F[3, 1,576] = 3.64, *P *= .012). Again, as predicted, pairwise comparisons showed that compared to exposure to the dominant narrative, exposure to the unaffordability and complexity counternarratives increased blame attributed to the medical industry (*P *= .029 and *P* = .016, respectively).

Significant differences across the four groups were also found regarding blame assigned to the federal government (F[3, 1,577] = 4.69, *P *= .0029). Pairwise comparisons showed that both the unaffordability and complexity counternarrative conditions significantly increased blame attributed to the federal government compared to the dominant narrative condition (*P* = .005 and .010, respectively), thus aligning with our H2 prediction.

#### Internal/External Causal Attribution

**Figure 2:**
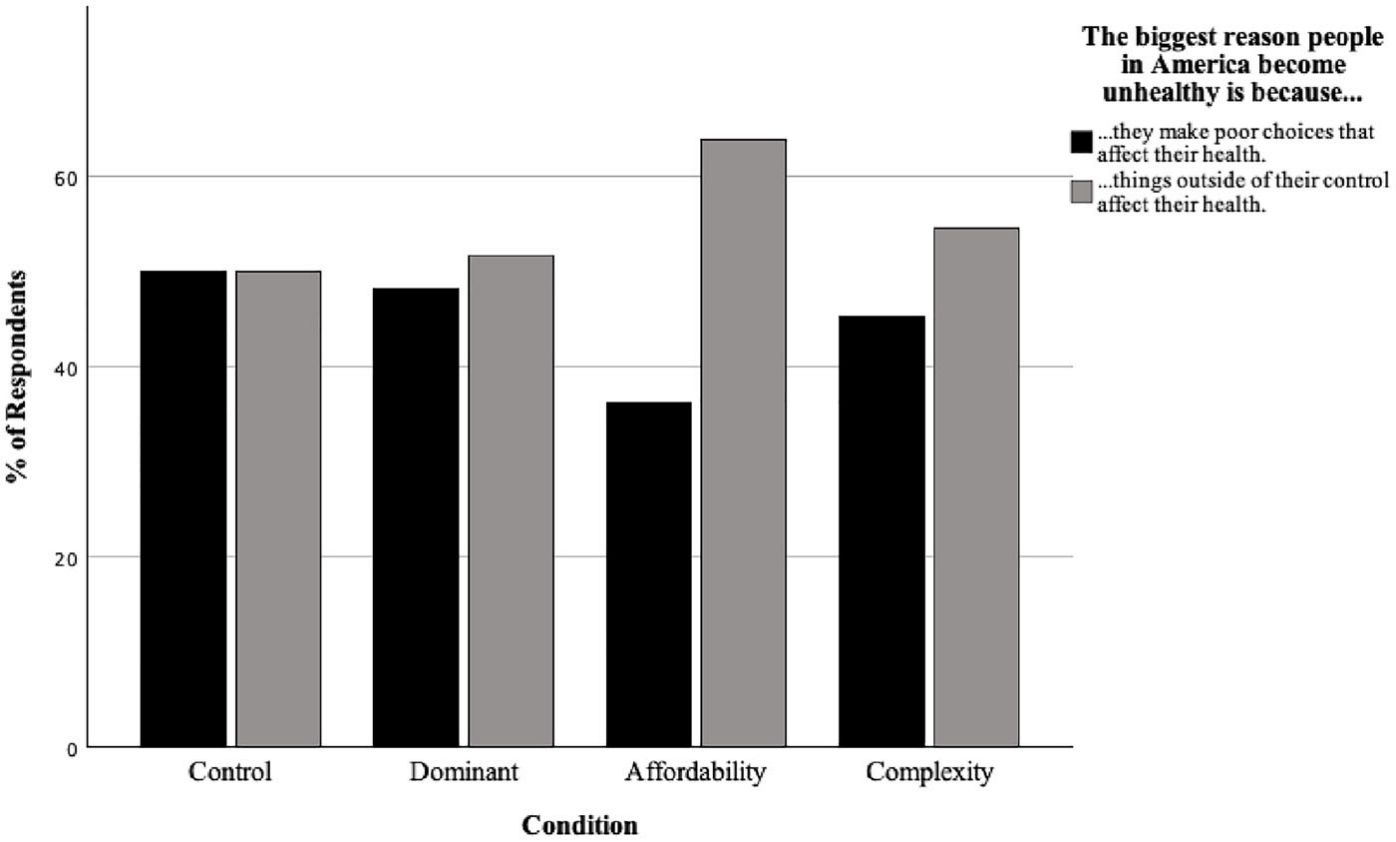
Causal Attributions for Health by Experimental Conditons [Colour figure can be viewed at wileyonlinelibrary.com]

Regarding whether participants attribute poor health in America to individual‐level poor choices or external factors outside of individuals’ control, results supported H2, as both of the counternarratives significantly increased attribution to external factors vs. internal factors, compared to the dominant narrative (Pearson chi‐squared(3) = 18.4979, *P* < .001). Results are reported in Figure 2.

### Exploratory Analyses: Subgroup Differences in Narrative Effects

We ran a series of regressions including an interaction term between experimental condition and each of the potential moderators in the model (eg, experimental condition × partisanship). This was repeated for each potential moderator and our primary outcomes. In all regressions, the no‐exposure condition was treated as the reference category. All regression tables are presented in Appendix C, and we display figures below showing the marginal effects from the models with statistically significant interaction terms.

#### Interaction Effects of Income and Experimental Condition

**Figure 3:**
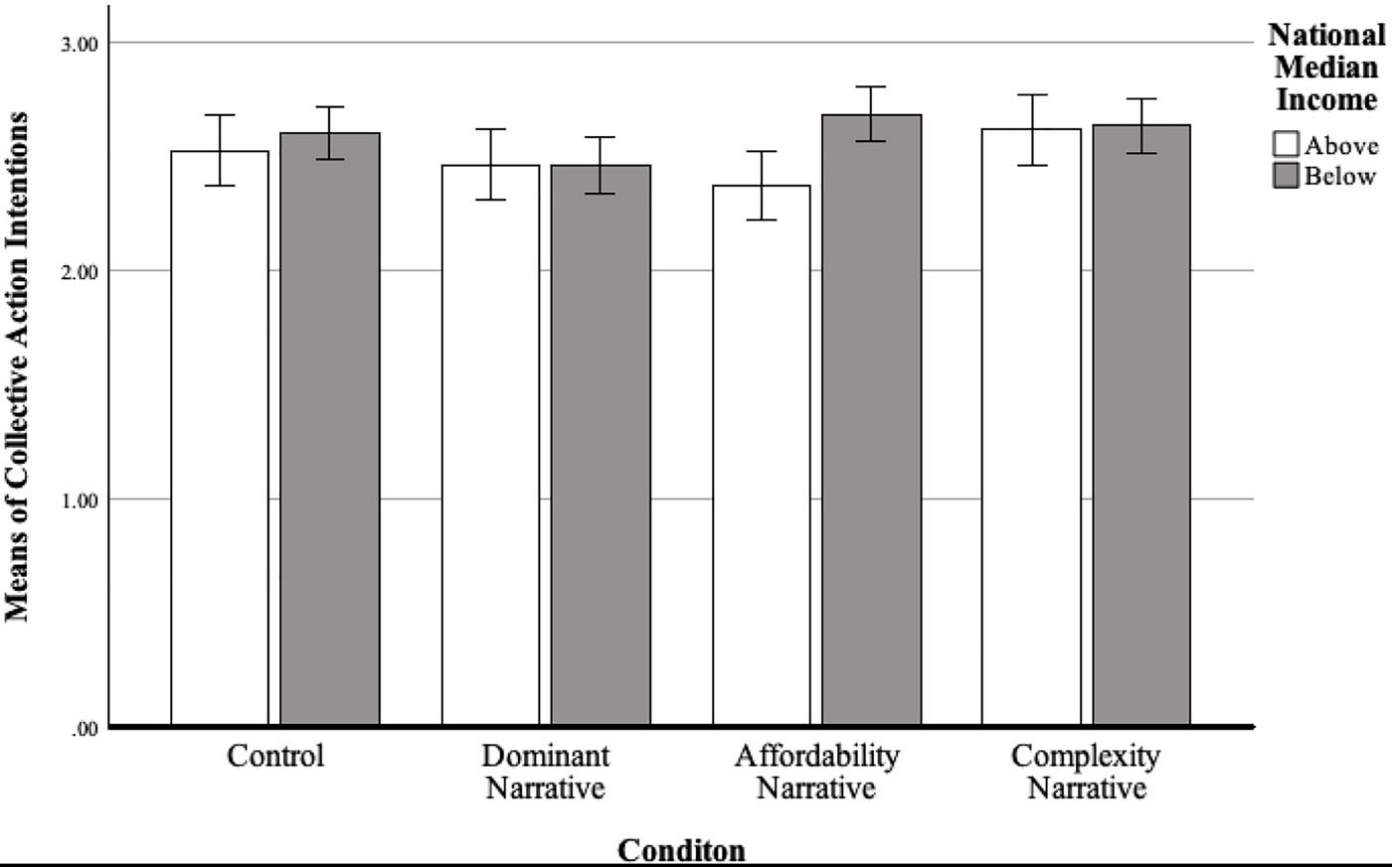
Interaction Effect of Income and Experimental Condition on Collective Action Intentions [Colour figure can be viewed at wileyonlinelibrary.com]

We observed a direct significant association between income and policy agenda setting (*β *= 0.22, SE = 0.09, *P *= .019), suggesting that those with income below the national median reported higher perceptions of the importance of policy action. However, there were no statistically significant interaction effects between the experimental conditions and income for policy agenda setting, meaning that the narratives did not affect respondents’ perceptions of the importance of policy based on their income. There were also no statistically significant interaction effects between the experimental conditions and income for social justice behavioral intentions. In contrast, a marginally significant interaction effect was detected between the experimental conditions and income on collective action intentions (*β *= 0.24, SE = 0.14, *P *= .090), such that the affordability counternarrative was particularly effective at boosting intentions to act among participants who report earning less than the national median household income (see Figure 3).

#### Interaction Effects of “Barriers to Health Care” and Experimental Condition

Results indicated there was a significant association between barriers to health care and policy agenda setting (*β *= 0.24, SE = 0.06, *P *< .001), with each increase in the number of barriers experienced associated with an increased perception of the importance of policy. However, there were no significant interaction effects between experimental conditions and barriers to health care on this outcome.

**Figure 4:**
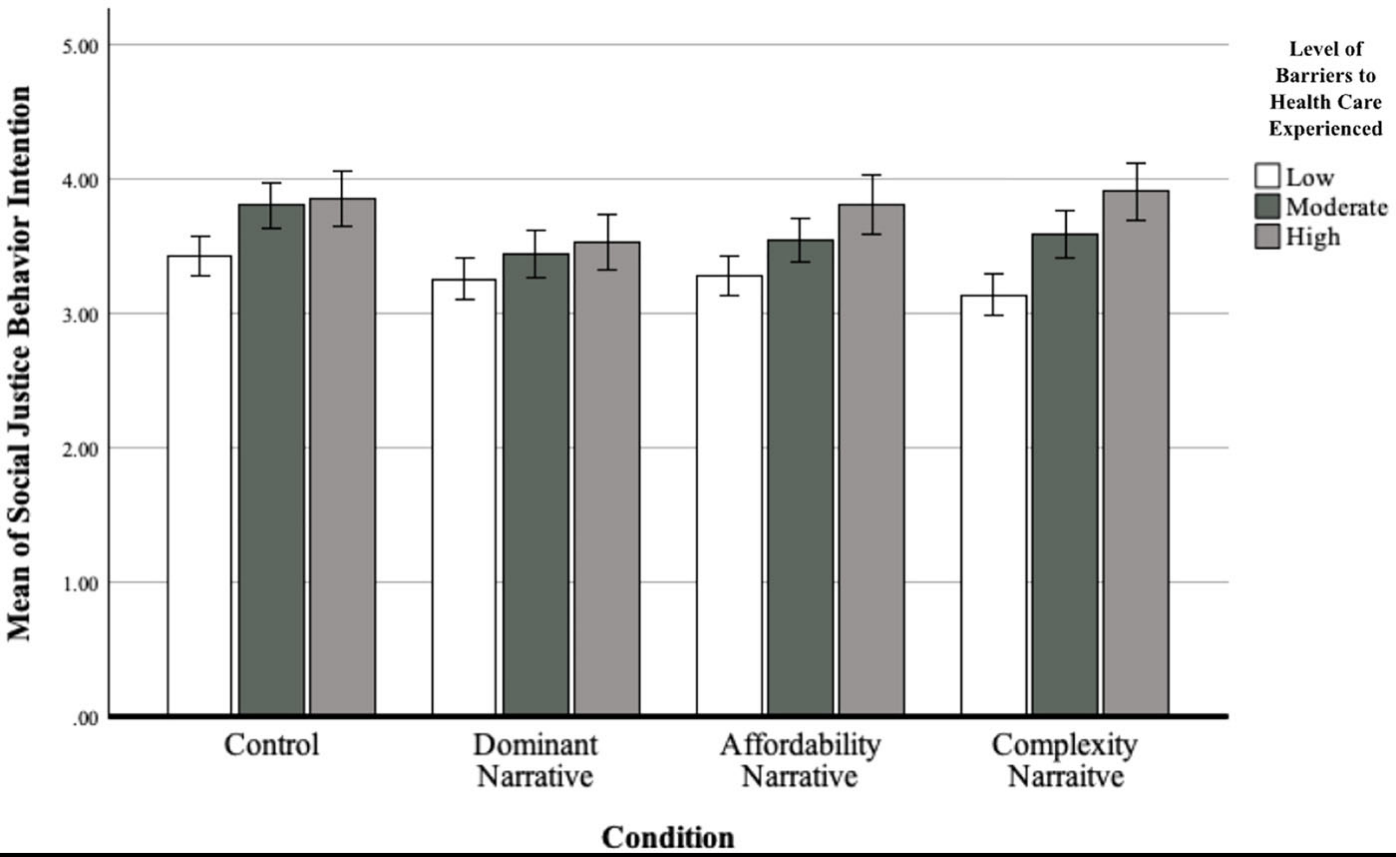
Interaction Effect of Cost Barriers and Experimental Condition on Social Justice Behavioral Intentions [Colour figure can be viewed at wileyonlinelibrary.com]

Similarly, barriers to health care significantly predicted collective action intentions (*β *= 0.29, SE = 0.06, *P *< .001) and social justice intentions (*β *= 0.24, SE = 0.02, *P *< .001), again in a positive relationship, with increasing barriers associated with more intentions. There was a marginally significant interaction effect of the complexity counternarrative and barriers to health care on social justice intention (*β *= 0.16, SE = 0.09, *P *= .09), suggesting that the effect of the complexity counternarrative on social justice intention may be stronger for those who have experienced more barriers (see Figure 4).

#### Interaction Effects of Partisanship and Experimental Condition

To test whether partisanship moderated the message effects, we estimated a model with partisanship entered as a variable with three categories: Democrats (including leaners) (*n *= 944), Republicans (including leaners) (*n *= 421) and Independents (*n *= 219), with interaction terms for Democrats and Independents, with Republicans as the reference category. These regressions showed that partisanship significantly predicted policy agenda setting such that both Democrats (*β *= 0.8, SE = 0.1, *P *< .001) and Independents (*β *= 0.39, SE = 0.14, *P *< .01) reported higher levels of perceived policy importance than did Republicans.

Regarding the other two outcomes, there was a significant relationship between identifying as a Democrat and higher collective action intentions (*β *= 0.49, SE = 0.11, *P *< .001) and higher social justice intentions (*β *= 0.58, SE = 0.12, *P *< .01). There was no significant association between the Independent group and collective action (*β *= 0.18, SE = 0.15, *P *= .24) or social justice intentions (*β *= 0.21, SE = 0.16, *P *= .2).

There were no significant interaction terms between partisanship and experimental condition on any of the three outcomes, indicating the messages did not differ in their impact based on participants’ partisanship. In sensitivity analyses (Appendix C, Table C.3), we estimated regressions with partisanship measured in alternative ways (first with Republicans vs. Democrats, then with leaners included in the main group). None of the models showed statistically significant interaction terms between partisan groups and experimental conditions.

## Discussion

The goal of our study was to apply a research framework to test what community organizers do in practice—expose dominant narratives in a particular policy arena (such as health care), which may constrain political possibilities, and seed counternarratives that resonate with people, to mobilize coalitions and create opportunities for political change (see, eg, Haapanen and colleagues^33^). While we tested messages within a survey‐based context, and not the real‐world setting of community organizing (as we discuss below), our results provide evidence reaffirming the intuition of organizers. Regarding how narratives shape intentions to participate in actions to improve the health care system (our primary outcomes), we found that compared to the dominant narrative, the complexity narrative *increased* intentions to participate in a few specific types of actions. Specifically, intentions to contact the media, attend a community forum, and attend a protest or rally were stronger after reading this counternarrative. Importantly, exposure to the dominant narrative *decreased* intentions to take collective action (eg, participating in community conversations) or engage in social justice behaviors (eg, talking to others about differences in health, engaging in activities that promote a more fair system). This is a concerning finding for organizers, given their present and historical model of success consists of working with racially and economically marginalized communities to confront structural injustices and win material changes specifically through taking collective action. That even brief exposure to the dominant narrative in the context of a research study can discourage such action is a striking and consequential finding.

Perceptions of causal attributions for problems with the health care system (our secondary outcomes) were also affected by narrative exposure. Specifically, we found that the dominant narrative about health care effectively does what health justice organizers argue it is designed to do: shift blame for health challenges from powerful institutions to individuals. This is a meaningful finding given that the dominant narrative fielded in our study focused predominantly on health care quality and innovation and included only a brief reference to “unhealthy behaviors.” This underscores how even subtle references to personal choices can perpetuate harmful myths of individual blame for structural problems.

However, encouragingly, we also found that the counternarratives grounded in the lived experiences and language of people closest to the problem (ie, organizing staff and community leaders at CHP) have potential to challenge prevailing narratives. In our survey, exposure to either counternarrative *increased* respondents’ perceptions of blame attributed to powerful actors, shifting responsibility from individuals to institutions. Notably, when we tested how narratives influenced respondents’ blame attribution on external vs. internal factors, we found that the unaffordability counternarrative was particularly effective in shifting blame toward systems, and reducing blame placed on patients.

Overall, our results suggest narrative strategies anchored in the core tenets of community organizing and RCN have the potential to influence people's behavior and attitudes. The alternative narratives used in this project shared common elements, including a shared values statement, identifying a clear antagonist or cause of the problems, offering an alternative vision, and including a call to action. A growing body of evidence suggests these communication emphases, that have become routine in organizing practice, can indeed shift attitudes and collective action intentions.^14^, ^15^

Additionally, despite the high degree of political polarization surrounding health policy generally,^55^ we did not see evidence of differential impact of the messages based on partisanship. While Democrats had higher intentions to take action to improve health care, they were no more responsive to the narratives than were Republicans. This is an important lesson that narratives rooted in the shared harms of the health care system might motivate the public, regardless of political predispositions. Indeed, recent public opinion work has shown that addressing health care costs is a bipartisan priority.^47^ Of course, which specific policy levers are the most appropriate for doing so is where partisan gaps are likely to emerge, as the ongoing federal and state political landscape surrounding health policy (ie, Medicaid cuts) makes clear.

### Limitations

There are a few important limitations in our study. First, the study sample (from Prolific) is not recruited to be nationally representative, although it was diverse in the dimensions important to this study. Second, we only delivered a single exposure to the narratives. Alternative designs delivering the narratives in multiple ways over time, and in multiple modes (ie, as news stories, speeches, interpersonal interactions), would more closely approximate the true context of organizing. As a related point, we only measured attitudinal and behavioral intention measures, not actual behavior change. Longitudinal designs and multiple narrative exposures would be important to evaluate whether the findings hold up in a more real‐world context. While measuring past behavior would have provided additional insight into whether self‐reported actions match reality (ie, social desirability bias may have elevated overall levels of behavioral intentions), random assignment makes it unlikely that such bias systematically differed across our conditions. Third, given we were drawing from a national sample of survey participants, we could not specify a specific campaign action in our narratives (eg, a real event, rally, or legislation under consideration). Therefore, the influence on action intentions may have been weakened as there was no specific action recommended in the narratives. Nonetheless, the statistically significant findings suggest that with more exposures and more specific actions described, we would be likely to see stronger narrative effects.

These are important directions for future research, including the evaluation of community organizing activities in practice,^56^, ^57^, ^58^ not only in a survey‐based simulation. Such work would also need to establish whether and how the counternarratives can be rooted in multiple arenas (ie, within media, government, cultural institutions, research) to develop narrative power at a scale that is beyond a single communication strategy and how this narrative power can shape policy goals and advance change within and across organizations (see, eg, Dorfman and colleagues^2^ and Han and colleagues^59^). Consistent with Haapanen and colleagues^33^, it is crucial to recognize that programmatic narrative interventions are limited in their capacity to produce meaningful social change when deployed in isolation from the larger constellation of community organizing processes. As such, they should be paired with broader strategies of cultivating sustained engagement in organizations that foster sociopolitical development and collective action.

### Implications for Research and Practice

The results of our study suggest counternarratives about the health care system have the potential to shift people's attitudes and beliefs about who is responsible for poor outcomes; we also offer a partnership model for health researchers to consider. Community organizers, who build power through developing counternarratives mobilizing support for equitable, transformative policies, can be important partners in pursuing empirical research to inform narrative strategy. As others have also argued (see, eg, Minkler and Wakimoto^60^ and Tattersall and Stears^61^), in these collaborations, researchers can ground their research questions and methodology in the experience and stories of people deeply impacted by the system. Such collaborations would ideally produce research projects that are more responsive to people's material conditions than can research agendas crafted by academics in isolation. A similar case has recently been made (in this journal) for more partnerships between community organizers and health departments.^62^

Finally, community organizers and health justice advocates can use these findings in their communication strategies that are one component of their broad set of tactics in producing change surrounding the increasing costs and complexity of health care. These research findings, especially if combined with other efforts that might confirm and expand upon this study, suggest that messaging approaches that give voice to the experiences of impacted communities can be an important mechanism for shifting narrative power.

## Funding/Support

This research was supported by the Robert Wood Johnson Foundation (grant 79754).

## Conflict of Interest Disclosures

The authors have no conflicts of interest to disclose.

## Supporting information



Supplementary Appendix (Online)
