# Supplementary material for: Participatory Research to Build Narrative Power: Results From Survey Research to Support Community Organizing for Health Justice and Equity
Source: Milbank Q. 2026 Jun 3:10.1111/1468-0009.70101. Online ahead of print. doi: 10.1111/1468-0009.70101 (PMC13559974; doi:10.1111/1468-0009.70101)
Supplement: Supplementary file 1 [file MILQ-9999-0-s001.docx]

**Supplementary Appendix (Online)**

**Appendix A: Qualitative Interview Themes**

*Below is a copy of the Emerging Themes Table the study team shared with CHP communications team organizers following Phase 1 of our research project.*

In **Phase 1** of our research project, we conducted interviews with 7 members of the organizing team. The major themes from our analysis are listed below, along with a description. In the next phase, we will be crafting *narrative elements* from both these insights and your collective ongoing narrative power work (i.e., identifying messages with strong narrative power potential).

Please add +’s to indicate how much the theme resonates with you in the “Leader reactions” column (e.g., + = resonates slightly;  ++ = resonates moderately, +++ = resonates a lot).

| **Emerging themes** | **Description** | **Leader reactions** |
| --- | --- | --- |
| Social network & care for community  motivates involvement with CHP | Leaders are drawn to CHP’s organizing work and values, in part, due to **witnessing their loved ones and family members experience challenges with the health system,** and are compelled to do something to change the conditions facing their communities. | +++  +  +++  +++  ++  +++  +++ |
| **Major challenges with the healthcare system** | | |
| Fragmentation and complexity places stress / burden on patients and families | When care delivery services are not streamlined, and different parts of the system (e.g., providers and insurance companies) are not interconnected, **patients bear the burden of coordinating the logistics of their own care** while simultaneously dealing with injury and illness. | +++  +++  +++  +++  +++  +++  +++ |
| Unaffordability | The enormous cost of health care services - made worse by a **lack of price transparency** and persistent confusion around prices - has created a tiered system in which money is the primary determinant of access to and quality of care. | +++  ++  +++  ++++++++++++++++++++++++++  ++++∞  +++  +++ |
| Disempowerment & dehumanization | Leaders describe feeling that they **have no *choice* or *autonomy* to make decisions that are best for themselves or their family**. Instead, they are at the will of the system, limited by what they can pay, what they are forced to sacrifice, and how much energy they can expend advocating or fighting for themselves.  **Doctors see patients** not as the whole person, but **as a “diagnosis,” “deficit,” or something to be “fixed.”**  This theme also includes “horror stories” of people forced to make life-or-death decisions because of inhumane, exploitative and extractive practices of the system. | ++  ++  ++  +++  ++  +++  ++ |
| Systemic oppression permeates the health care system (e.g., medical racism, classism, and general lack of cultural competence) | The current healthcare system is directed by systemic oppression, which creates **discriminatory experiences for non-dominant group members**. For example, leaders shared that when navigating the health system, people are treated differently based on their racialized identity, English speaking ability, perceived class status, and income. | +++  +++  +++  +++  ++  +++  +++ |
| Tensions around trust | Despite having *some* confidence in individual providers, it is extremely **difficult to have full faith in healthcare *institutions****.* The lack of confidence in the healthcare system is tied closely to the profit motive: it is hard to trust the word of hospitals and insurance companies, given that **these institutions’ primary motivation and goal is turning profit, not caring for people.** | **+++++**  **+++**  **+++**  **+++**  **++**  **++**  **++** |
| Complexity | The system is incredibly complicated. Insurance plans are elaborate and difficult to understand; it isn’t clear how these plans map to hospitals and providers; the process for calculating bills is opaque; and often different parts of the system don’t “talk” to each other. **Patients pay the ultimate price for this complexity.** | +++  ++  +++  +++  ++++  +++  +++ |
| Limited Healthcare Access | Obtaining care is not straightforward, especially for poor and working-class families. Access to different types of care **varies based on insurance** (this is especially relevant for those on Medicaid). It can take months to see a doctor, and even then, it might not be the right doctor in the right modality. Oftentimes, **it is hard to simply access information needed to make the best decision for one’s self or family.** | ++  +++  +++  ++  +  +++  ++ |
| **Root causes of health care system challenges** | | |
| The profit motive | All components of the system are incentivized to **maximize financial gain and reduce costs**, even at the expense of patients. From the leaders’ perspective, the system’s primary goal is to make money, and serve a **broader capitalist nation** that is always thinking about profit. Instead of getting care that respects the needs of patients, people get services that churn the most profit, and involve the least amount of time. | +++  +++++  ++++  +++++  +++  ++ |
| Media narratives obscure problems | News media, TV shows and pop culture depict the healthcare system in an unrealistic manner. For example, **advertisements make it look “easy” and even pleasant to access care**; the care depicted looks coordinated; pop culture implies that healthcare in the US is universal (e.g., experiences of uninsured/underinsured people are not highlighted); and we hardly see examples of patients getting turned away because they cannot afford care. | ++  ++  ++  +++  +++  +++  +++ |
| **Attributes of a new system** | | |
| Holistic ecosystem of care | A **healthy community is one that is interconnected,** where people do not live isolated lives in which work is at the center. In this context, the healthcare system must be one component of a broader ecosystem of care/wellbeing that includes housing, food, climate justice, etc. This ecosystem must prioritize **collective outcomes > individual behaviors.** | ++++++++  ++++  +++  +++  +  +++  +  +++++  ++ |
| Personalized care and genuine 1:1 connection | In an ideal system, the current power dynamic that arises between people “seeking” vs. “giving” care will be dismantled. Instead, providers will reflect the communities they serve, and be resourced/supported such that they can **spend adequate *time* with those in need of care**, getting to know them as *people*, not just “patients.” | +++  +++  +++  +++  ++  +++  ++ |
| Patients are empowered by transparency and reduced complexity | **People seeking care should know all their options** - including final costs - upfront, **and be empowered to make their own decisions**, armed with complete information. Furthermore, in an ideal system, there will be more deference to patients; providers will **trust the patient's body and knowledge as a primary source of knowledge** to direct the care, and people should feel involved in the process. | +++  +++++  ++++++++  ++++  +++++  +++ |

**Appendix B: Full Narratives**

*Dominant narrative*

The U.S. healthcare system - including the hospital, insurance and pharmaceutical industries -

is widely recognized as the leading care delivery system in the world. Our nation’s state-of-the-

art hospitals and centers are world leaders in innovation and quality. We have access to the

most advanced technology and top-tier physician leaders from across the globe.

However, our health system is struggling in the face of significant regulatory burdens as well as sicker communities, because of rising rates of disease borne from obesity and unhealthy

behaviors. Even as these issues raise the cost of providing care, our health systems continue to

keep patients at the center. Through mergers and acquisitions and robust investments in the

latest technology, research and infrastructure, our hospitals are able to better coordinate far-

reaching systems of care that ensure no patient is left behind.

In the next decade, health systems will continue to evolve and transform, redesigning care

delivery to provide consumers with convenient, cost-effective and technologically advanced

services. Many innovative drugs and treatments are on the horizon, because of our investments

in research and development.

Although there is some push for increasing the role of government in the provision of health

services, we are confident that publicly-managed healthcare would increase costs and limit

access to care. This would hinder access to the high-quality, innovative care our patients -

especially those in need of complex services - have come to expect and deserve.

Through community benefit investments, millions spent annually on uncompensated care, and

partnerships with philanthropic organizations serving under-resourced communities, America’s

hospitals take care of our communities. Our healthcare system is working for you

*Unaffordability narrative*

All of us, no matter where we live, how much money we make, or whether we are Black,

white, Asian, Indigenous, or Latino, deserve access to affordable, timely, and high quality health

care when we are sick. Easy access to affordable, comprehensive medical care should be

possible for everybody.

But the reality is that most people and families just can’t afford the medical care that is available

to us. When we get sick, which is already stressful enough, thinking about medical bills or what

we would need to sacrifice to afford treatment increases our stress. Sometimes we put off

getting help, which makes our health even worse. It makes me feel like I’m just a dollar sign in

this expensive system, not a whole person.

These issues with the healthcare system have real impacts on our day-to-day lives. When I

have to deal with the system, I feel upset and powerless. When my kids or family get sick, I get

frustrated, even when I know it’s not their fault. Sometimes I don’t even let my kids play freely

because I’m scared they will get hurt, and if that happens, we will have to see a doctor and

spend thousands of dollars we don't have. It makes me want to avoid health care entirely.

These problems are not accidental. Executives working for the hospital, insurance, and

pharmaceutical industries have made this system the way it is to benefit them, not to take care

of everyday people like us. They treat healthcare just like any other business in the U.S., trying

to make as much money as possible. These executives are making millions of dollars a year,

and I can barely afford to see the doctor.

They want us to blame ourselves for being sick, so we won’t hold them responsible for how

complex and wasteful the health care system is. Corporate health care takes public money that

could be spent to keep us and our communities healthy and instead uses it to drive up their

profits.

I deserve a health care system that sees me as a whole person - we all do. We should be able

to get the care we need, when we need it, and focus on getting better instead of worrying about

money. Hospitals and clinics would be just one part of a healthy community, alongside adequate

housing, food, and a healthy environment.

This vision can become a reality. Contact your local elected leaders to demand health care that

works for all of us, and leaves nobody out. Connect with organizations in your community that are making progress everyday by applying pressure so healthcare organizations will put our care over their profits. Together, we have the power to make change so we

can all get the care we need, no matter what’s in our wallet, whether we’re young or old, Black

or white, native or newcomer.

*Complexity narrative*

All of us, no matter where we live, how much money we make, or whether we are Black, white,

Asian, Indigenous, or Latino, deserve access to affordable, timely, and high quality health care

when we are sick. Easy access to affordable, comprehensive medical care should be possible

for everybody.

But the reality is that even though a lot of health care workers want to help patients get the

health care they need, the systems they operate in are disconnected and too complicated. The

patient is left with the time-consuming stress of coordinating between so many people - the

doctors, the insurance, the pharmacy, the specialists. There is nobody to help us put all the

pieces together - nobody but us advocating for our best interests. It makes me feel like I’m just a

number in this stressful system, not a whole person.

These issues with the healthcare system have real impacts on our day-to-day lives. When I

have to deal with the system, I feel upset and powerless. When my kids or family get sick, I get

frustrated, even when I know it’s not their fault. I don’t even let my kids play freely because I’m

scared they will get hurt, and if that happens, I’ll have to deal with this complicated system and

spend time - that I don’t have - advocating for my kids’ needs. It makes me want to avoid health

care entirely.

These problems are not accidental. Executives working for the hospital, insurance, and

pharmaceutical industries have made this system the way it is to benefit them, not to take care

of everyday people like us. They treat healthcare just like any other business in the U.S., trying

to make as much money as possible. These executives are making millions of dollars a year,

and I can barely afford to see the doctor.

They want us to blame ourselves for being sick, so we won’t hold them responsible for how

complex and wasteful the health care system is. Corporate health care takes public money that

could be spent to keep us and our communities healthy and instead uses it to drive up their

profits.

I deserve a health care system that sees me as a whole person - we all do. We should be able

to get the care we need, when we need it, and focus on getting better instead of worrying about

how to put all the pieces together by ourselves. Hospitals and clinics would be just one part of a

healthy community, alongside adequate housing, food, and a healthy environment.

This vision can become a reality. Contact your local elected leaders to demand health care that

works for all of us, and leaves nobody out. Connect with organizations in your community that are making progress everyday by applying pressure so healthcare organizations will put our care over their profits. Together, we have the power to make change so we can all get the care we need, no matter what’s in our wallet, whether we’re young or old, Black

or white, native or newcomer

**Appendix C: Regression Models with Interaction Terms**

**Table C.1.** Moderation analysis of barriers to healthcare  on the relationship between message exposure and agenda setting, collective action intentions, and social justice behavioral intentions.

|  | Agenda Setting | Collective Action | Social Justice |
| --- | --- | --- | --- |
| Variable | b(SE) | b(SE) | b(SE) |
| Dominant | -.021(.09) | -.059(.10) | -.196(.10) |
| Affordability | -.043(.09) | -.017(.09) | -.187(.10) |
| Complexity | -.044(.09) | -.045(.09) | -.314(.10)** |
| Barriers | .242(.06)** | .292(.06)** | .236(.02)** |
| Dominant x Barriers | -.111(.08) | -.088(.09) | -.096(.09) |
| Affordability x Barriers | .002(.08) | .003(.09) | .031(.09) |
| Complexity x Barriers | -.013(.08) | .114(.09) | .158(.09)^a^ |
| R^2^ | .04 | .07 | .05 |
| F(df) | 8.32(7)** | 15.49(7)** | 12.02(7)** |

*Note.*Barriers to healthcare were measured categorically 0 = low experience with healthcare barriers (0-2), 1 = moderate experience with healthcare barriers(3-5), 2 = high experience with healthcare barriers (5<). ^a^ = *p* < .10, * = *p* < .05, ** = *p* < .01

**Table C.2.** Moderation analysis of income on the relationship between message exposure and agenda setting, collective action intentions, and social justice behavioral intentions.

|  | Agenda Setting | Collective Action | Justice 1 |
| --- | --- | --- | --- |
| Variable | b(SE) | b(SE) | b(SE) |
| Dominant | .018(.11) | -.060(.11) | -.188(.12) |
| Affordability | -.063(.10) | -.154(.11) | -.216(.12)^a^ |
| Complexity | -.069(.10) | .094(.11) | -.116(.12) |
| Income | .219(.09)* | .078(.10) | .087(.11) |
| Dominant x Income | -.163(.13) | -.079(.14) | -.085(.15) |
| Affordability x Income | .056(.13) | .238(.14)^a^ | .119(.15) |
| Complexity x Income | .031(.13) | -.059(.14) | -.094(.15) |
| R^2^ | .010 | .006 | .006 |
| F(df) | 3.38(7) | 2.41(7) | 2.29(7) |

*Note.* Income was recoded categorically so that 0 = income above the national median household income, 1 = income below the national median household income, . ^a^ = *p* < .10, * = *p* < .05, ** = *p* < .01

**Table C.3.** Moderation analysis of partisanship^b^ on the relationship between message exposure and agenda setting, collective action intentions, and social justice behavioral intentions.

|  | Agenda Setting 1 | Agenda Setting 2 | Agenda Setting 3 | Collective Action 1 | Collective Action 2 | Collective Action 3 | Justice 1 | Justice 2 | Justice 3 |
| --- | --- | --- | --- | --- | --- | --- | --- | --- | --- |
| Variable | b(SE) | b(SE) | b(SE) | b(SE) | b(SE) | b(SE) | b(SE) | b(SE) | b(SE) |
| Dominant | -.091(.13) | -.078(.11) | -.078(.12) | .120(.15) | .001(.131) | .001(.133) | -.053(.162) | -.224(.14) | -.224(.141) |
| Affordability | .128(.12) | .043(.11) | .043(.11) | .133(.15) | .008(.128) | .008(.13) | .019(.155) | -.126(.137) | -.126(.138) |
| Complexity | -.093(.12) | -.119(.11) | -.119(.11) | .093(.14) | .03(.125) | .03(.127) | -.146(.151) | -.241(.134)^a^ | -.241(.135)^a^ |
| Democrat | .888(.11)** |  |  | .555(.13)** |  |  | .687(.132)** |  |  |
| Dominant x Democrat | -.024(.15) |  |  | -.248(.18) |  |  | -.196(.192) |  |  |
| Affordability x Democrat | -.183(.15) |  |  | -.150(.18) |  |  | -.186(.187) |  |  |
| Complexity x Democrat | .045(.15) |  |  | -.001(.17) |  |  | -.089(.183) |  |  |
| Democrat (plus leaners) |  | .802(.09)** | .802(.10)** |  | .488(.108)** | .488(.11)** |  | .578(.116)** | .578(.117)** |
| Independent |  |  | .391(.14)* |  |  | .182(.154) |  |  | .211(.163) |
| Dominant x Democrat (plus leaners) |  | -.021(.13) | -.021(.14) |  | -.146(.156) | -.146(.159) |  | -.004(.167) | -.004(.168) |
| Affordability x Democrat (plus leaners) |  | -.08(.13) | -.08(.14) |  | -.009(.154) | -.009(.156) |  | -.027(.164) | -.027(.166) |
| Complexity x Democrat (plus leaners) |  | .11(.13) | .105(.14) |  | .064(.152) | .064(.154) |  | .079(.162) | .079(.163) |
| Dominant x Independent |  |  | -.13(.20) |  |  | -.303(.227) |  |  | -.285(.242) |
| Affordability x Independent |  |  | -.186(.19) |  |  | -.077(.215) |  |  | -.01(.229) |
| Complexity x Independent |  |  | .177(.20) |  |  | -.028(.22) |  |  | .219(.234) |
| R^2^ | .200 | .182 | .154 | .054 | .056 | .056 | .074 | .078 | .076 |
| F(df) | 37.42(7) | 43.24(7) | 26.01(11) | 8.40(7) | 11.44(7) | 8.43(11) | 11.91(7) | 16.28(7) | 11.65(11) |

*Note.* ^b^For each outcome, in the first model, partisanship consisted of two categories: Democrats and Republicans; in the second model, the categories of Democratic and Republican also included participants who identified as Independent but leaned toward one party or the other (“leaners”); in the third model, partisanship included three categories: Democrats (including leaners), Republicans (including leaners), and Independents. For all models, Republican was used as the reference category for partisanship.

^a^ = *p* < .10, * = *p* < .05, ** = *p* < .01
